# Preparation of NASICON-Type Nanosized Solid Electrolyte Li_1.4_Al_0.4_Ti_1.6_(PO_4_)_3_ by Evaporation-Induced Self-Assembly for Lithium-Ion Battery

**DOI:** 10.1186/s11671-016-1768-z

**Published:** 2016-12-15

**Authors:** Xingang Liu, Ju Fu, Chuhong Zhang

**Affiliations:** State Key Laboratory of Polymer Materials Engineering, Polymer Research Institute, Sichuan University, Chengdu, 610065 China

**Keywords:** Nano-LATP, Evaporation-induced self-assembly (EISA), Solid electrolyte, Lithium-ion battery

## Abstract

A simple and practicable evaporation-induced self-assembly (EISA) method is introduced for the first time to prepare nanosized solid electrolyte Li_1.4_Al_0.4_Ti_1.6_(PO_4_)_3_ (LATP) for all-solid-state lithium-ion batteries. A pure Na^+^ super ion conductor (NASICON) phase is confirmed by X-ray diffraction (XRD) analysis, and its primary particle size is down to 70 nm by optimizing evaporation rate of the solvent. Excellent room temperature bulk and total lithium-ion conductivities of 2.09 × 10^−3^ S cm^−1^ and 3.63 × 10^−4^ S cm^−1^ are obtained, with an ion-hopping activation energy as low as 0.286 eV.

## Background

All-solid-state batteries using ceramic electrolytes have attracted great interests due to their good chemical stability, high ionic conductivity, and superior safety which have been considered as the ultimate safe batteries [1]. Li_1+*x*_Al_*x*_Ti_2−*x*_(PO_4_)_3_ (LATP) is one of the fastest lithium-ion-conducting ceramics with a Na^+^ super ion conductor (NASICON) type structure [2], offering great potential as solid electrolytes applied in all-solid-state lithium-ion batteries (LIBs) [3].

Particle size plays an important role on conductivity of crystalline electrolytes, and usually reduction on particle size favors improved ionic conductivity [4, 5]. There are various methods to synthesize LATP such as solid state reaction, co-precipitation, sol-gel, and melting-quenching methods [6–9]. High temperature approaches including solid state reaction and melting-quenching usually involve high energy consumption and loss of stoichiometric lithium associated with impurities in the final product. In addition, among the above methods, sol-gel method is the only way to obtain nanocrystalline LATP at a moderate temperature. However, as a crucial step of sol-gel method, controlling hydrolysis of titanium salt is relatively complex and time-consuming. Therefore, in this contribution, we investigated a simple evaporation-induced self-assembly (EISA) method suitable for large-scale production of nanosized LATP (*X* = 0.4) with high ionic conductivity, which employs ethanol (EtOH) as solvent to avoid the hydrolysis of titanium salt. This work may also offer a new strategy for fabrication of other LATP solid electrolytes with varying *x* values.

## Methods

Ethanol soluble LiNO_3_•H_2_O, AlCl_3_, TiCl_4,_ and H_3_PO_4_ were used as starting materials. All chemicals were of analytical grade and used as received, without further purification. They were accurately weighed with the stoichiometric ratio as in Li_1.4_Al_0.4_Ti_1.6_(PO_4_)_3_. TiCl_4_ and AlCl_3_ were first dissolved in EtOH and magnetically stirred for 30 min. Then LiNO_3_•H_2_O and H_3_PO_4_ were added to the solution under vigorous stirring for another 2 h. The homogenous sol solution was poured into open Petri dishes and heated at various temperatures (30, 50, and 70 °C) in air allowing the solvent to evaporate until viscous sol-gel was formed. We therefore denominated the samples by evaporation temperature, e.g., LATP-30 meaning EtOH evaporated at 30 °C. The sol-gel was further aged at 100 °C for 6 h to become the dry LATP precursor. Such dried gel was subsequently pyrolyzed in air at 300 °C for 3 h, followed by annealing at 800 °C for 2 h.

Thermogravimetric analysis (TGA) was performed on the dried gel (LATP precursors) at a heating rate of 10 °C min^−1^ in a temperature range of 30–800 °C under air atmosphere. The crystal structures of LATP powders prepared with various evaporation rate of EtOH were investigated by powder X-ray diffraction (XRD) on a Rigaku (SmartLab III) using Cu Kα radiation within 2*θ* = 10–70° with a step width of 0.06°. The microstructure and morphology were observed by field emission scanning electron microscopy (FESEM) (Quanta FEI, America). The as-prepared powders were pelletized by cold pressing at 200 MPa to a diameter and thickness of 13 and 1.3 mm, respectively. The pellets were sintered at 950 °C for 6 h in air and sputtered with Au on both sides as blocking electrodes for ionic conductivity measurements. AC impedance measurements were performed using an AutoLab 302–N impedance analyzer with an AC voltage of 5 mV over a frequency range of 10^−1^–10^5^ Hz.

## Results and Discussion

**Fig. 1:**
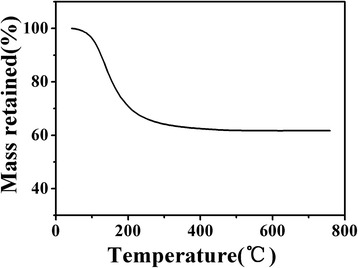
TGA curve of the dried gel for LATP precursors prepared by EISA method (heating rate 10 °C min^−1^, air atmosphere)

Figure 1 displays the TGA curve of the dried gel for LATP precursors. A sharp weight loss of about 35% occurs between 100 and 300 °C, as a result of decomposition of volatile components such as NO_3_^−1^, Cl^−1^, and H_2_O. While with increasing temperature, no further weight loss is observed. Therefore, based on the TGA result, in order to obtain final LATP powders, the dried gel was devised to a heat treatment procedure as described in the experimental section.

**Fig. 2:**
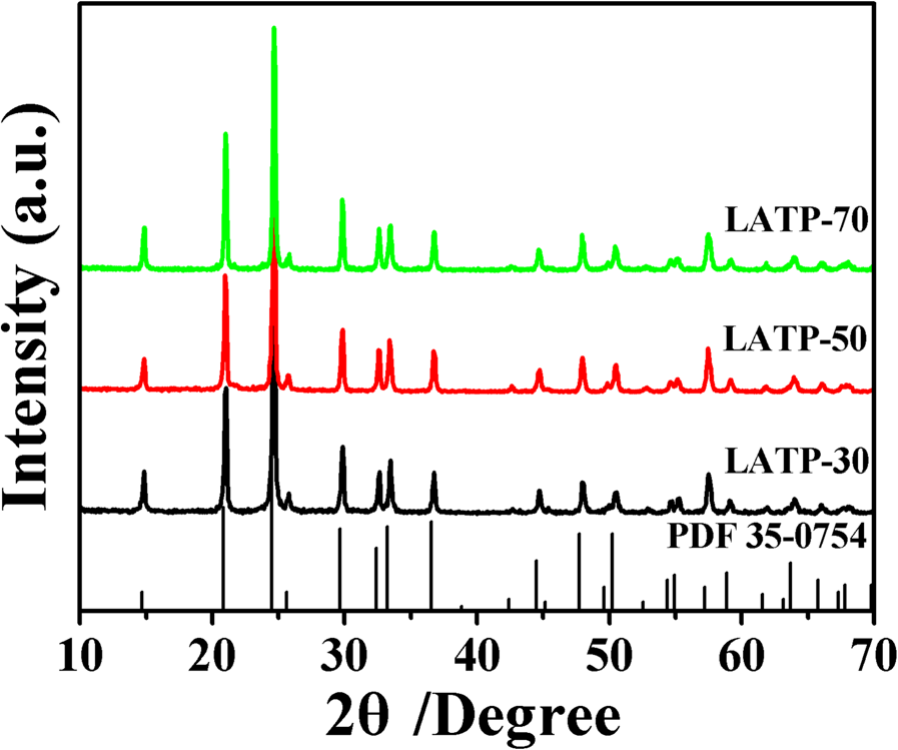
XRD patterns of LATP prepared with various evaporation rate of EtOH

X-ray diffraction patterns of the LATP powders prepared at various evaporation temperatures are shown in Fig. 2. The standard diffraction peaks of LiTi_2_(PO_4_)_3_ (JCPDS card No. 35-0754) are also indicated. Pure NASICON structured LATP is successfully obtained in all cases and there is no obvious difference on the crystal phase between these samples by varying the evaporation temperature. The lattice constants calculated from the XRD data are *a* = 0.8471 nm and *c* = 2.0763 nm, respectively, belonging to R-3c space group.

**Fig. 3:**
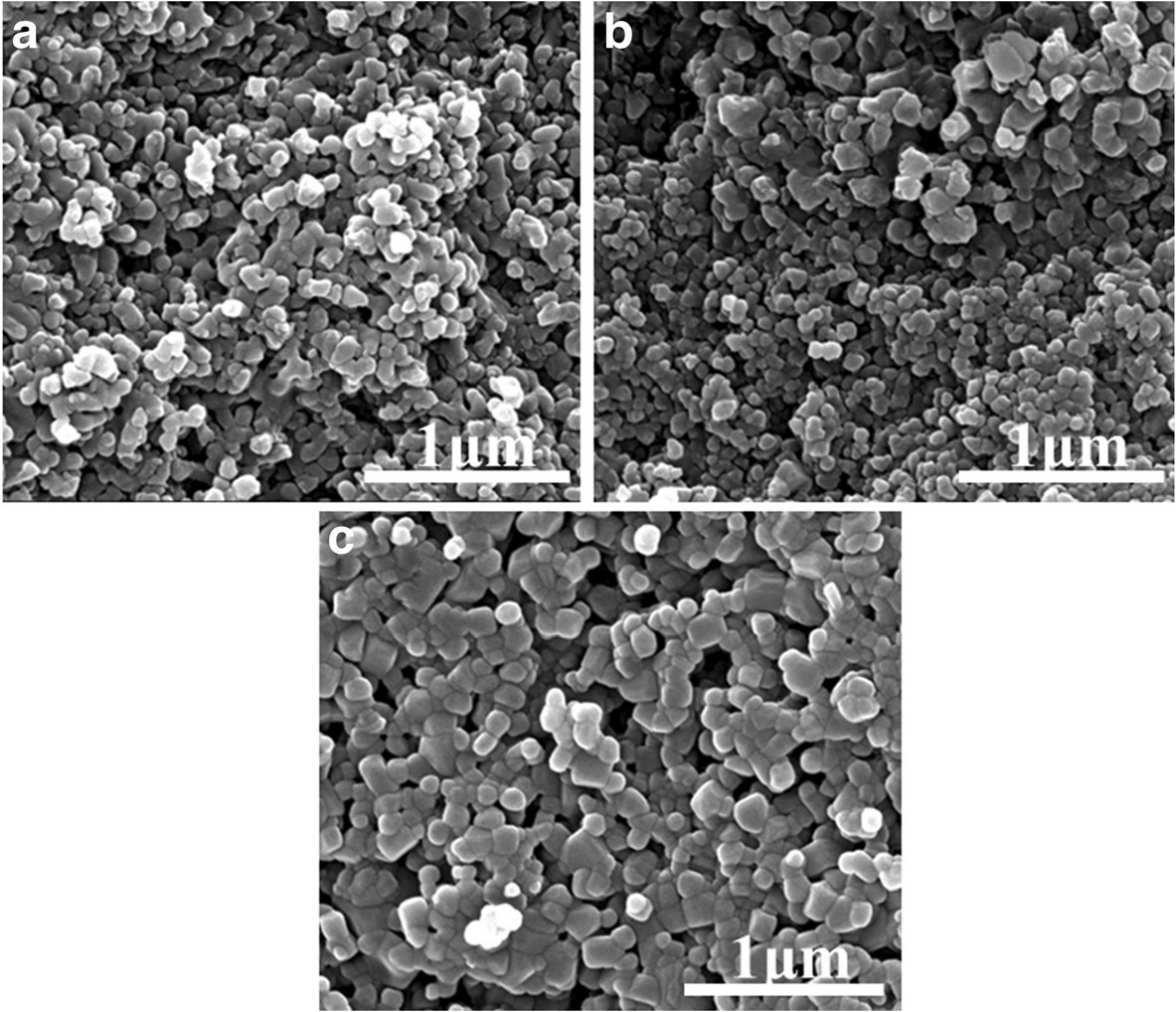
FESEM images of **a** LATP-30, **b** LATP-50, and **c** LATP-70 prepared with various evaporation rate of EtOH

**Fig. 4:**
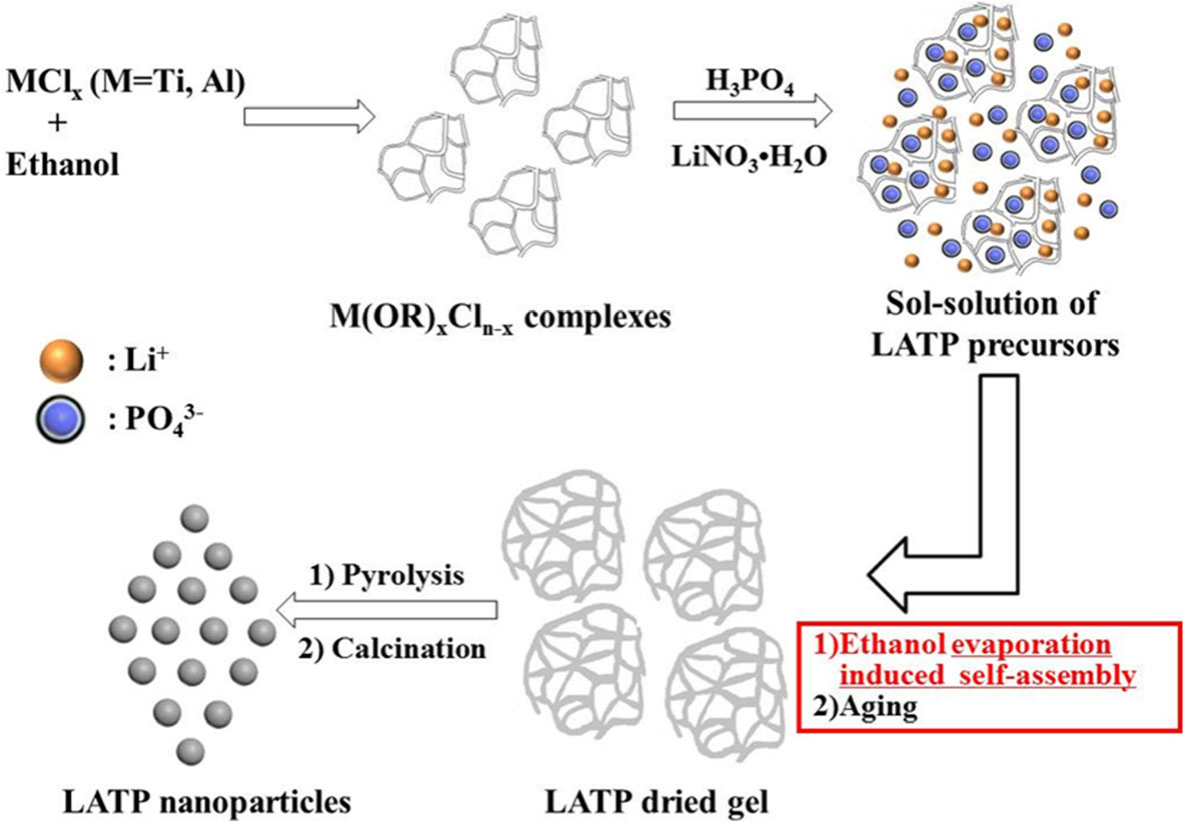
Schematic illustration of the synthesizing processes for LATP via EISA method

However, the SEM images clearly demonstrate that the evaporation rate of EtOH is crucial in determining the particle size of the LATP nanoparticles. As shown in Fig. 3, the average particle size of LATP-30, LATP-50, and LATP-70 are 70, 90, and 100 nm, respectively. The synthesizing process via the EISA method is schematically illustrated in Fig. 4. The so-called EISA process is an efficient approach to tune inorganic “polymerization” with the formation of a mesostructured template during the solvent evaporation, which has been widely applied to production of ordered porous materials or nanocomposites in the form of films, fibers, or powders [10, 11]. In this work, EISA is first developed to prepare nanocrystals of ceramic electrolyte LATP. When TiCl_4_ and AlCl_3_ are dissolved in EtOH, the metal chlorides react vigorously with EtOH to form complexes M(OR)_*x*_Cl_*n*−*x*_ (*n* = 3−4, *x* = 1−3), which can further self-assemble or polymerize to inorganic oligomers or even frameworks when the solvent EtOH is gradually removed. At the meanwhile, the Li^+^, PO_4_^3−^ ions dispersed in the sol solution deposit onto the M(OR)_*x*_Cl_*n*−*x*_ aggregates, which will be converted to LATP precursors after aging. Therefore, increase on the evaporation temperature promotes the crosslinking of M(OR)_*x*_Cl_*n*−*x*_, resulting in growth of the precursors thus bigger particle size of the final LATP powders.

**Fig. 5:**
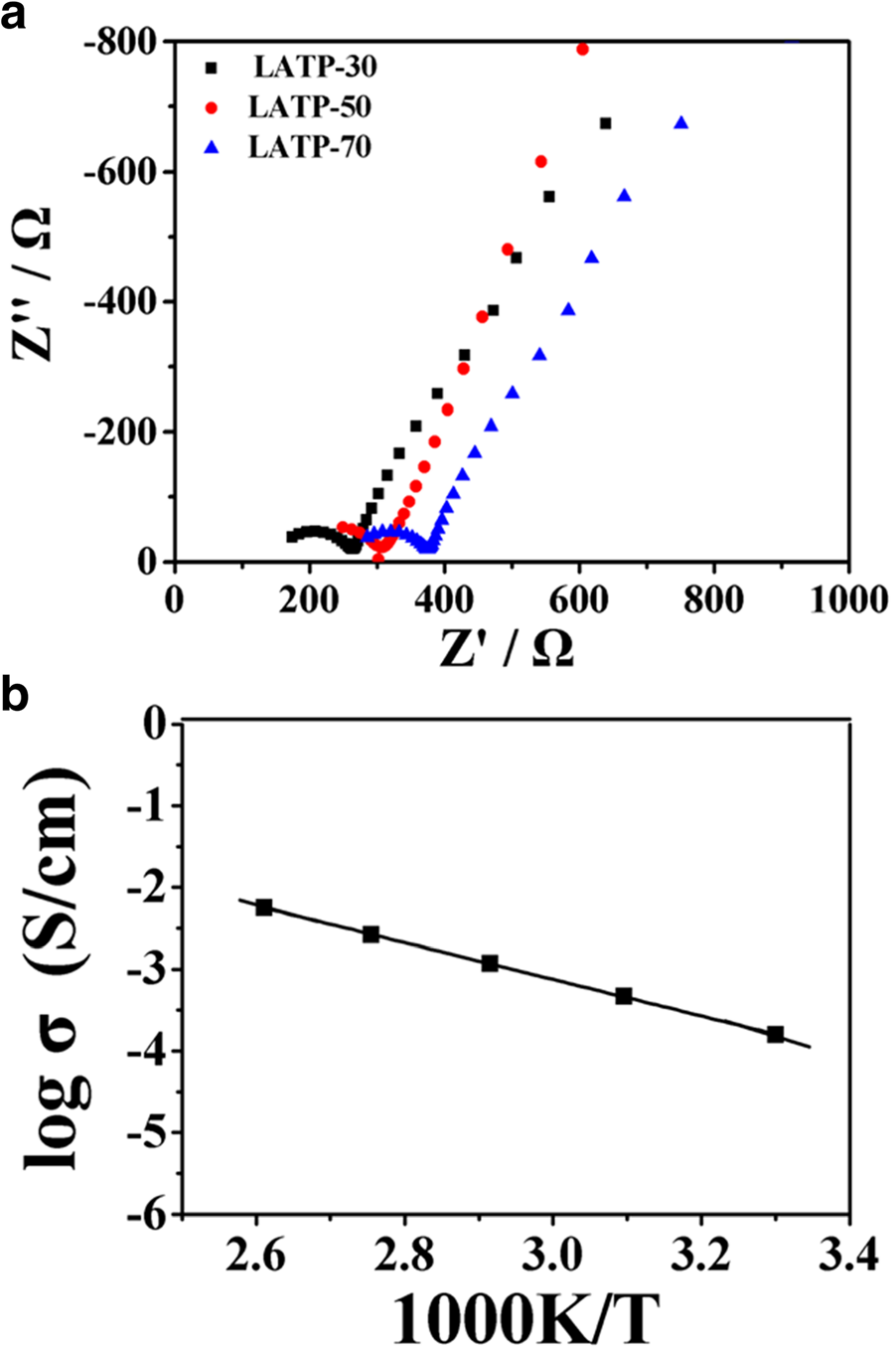
**a** Room temperature AC impedance plots of LATP prepared with various evaporation rate of EtOH (sputtered Au electrodes). **b** Arrhenius plots of total conductivity of the pelletized LATP-30 electrolyte

The room temperature Nyquist plots of LATP prepared with various evaporation rate of EtOH are demonstrated in Fig. 5a. Only one semi-circle at high frequencies along with an inclined spike at low frequencies is observed for all samples. As using blocking electrodes, the semi-circle corresponds to the transportation properties of Li^+^ in the ceramic electrolyte. The bulk resistance (*R*_b_) and total resistance (*R*_t_ = *R*_b_ + *R*_gb_) (*R*_gb_ refers to the grain boundary resistance) can be calculated from the left and right intercepts of the semi-circle with the real axis, respectively [12]. Thus, one can obtain the bulk conductivity (*σ*_b_) and total conductivity (*σ*_t_) using equation: *σ* = *l*/*R* · *S*, where *l* and *S* are thickness and area of the disk, respectively. As shown in Fig. 5a, both the *σ*_b_ and *σ*_t_ increase as the evaporation temperature decreases from 70 to 30 °C. The bulk and total conductivities of different LATPs at room temperature are summarized in Table 1. As can be seen, LATP-30 has the maximum room temperature *σ*_b_ and *σ*_t_ of 2.09 × 10^−3^ S cm^−1^ and 3.63 × 10^−4^ S cm^−1^, respectively.

**Table 1:** Average particle size and room temperature conductivities of LATP prepared with various evaporation rate of EtOH

Sample name	Average particle size (nm)	Bulk conductivity (*σ*_b_) (S cm^−1^)	Total conductivity (*σ*_t_) (S cm^−1^)
LATP-70	100	1.52 × 10^−3^	2.6 × 10^−4^
LATP-50	90	1.89 × 10^−3^	3. 3 × 10^−4^
LATP-30	70	2.09 × 10^−3^	3.63 × 10^−4^

The temperature dependence of the total conductivity of LATP-30 in the temperature range of 30–110 °C is shown in Fig. 5b. The plots of log(*σ*) against 1000/*T* are linear and well fitted by the Arrhenius equation as *σT* = *A*exp(−*E*a/*kT*), where *A* is the pre-exponential parameter, *k* is the gas constant, and *E*a represents the ion-hopping activation energy of the ceramic electrolyte [13]. The *E*a of LATP-30 sample calculated from the slope of linear fitted line is 0.286 eV. NASICON-structured lithium electrolytes usually have a constant *E*a of about 0.3 eV for the bulk, but the *E*a value at the grain boundary varies depending on the M^3+^ content and the degree of densification [14, 15]. The relative densities of the pressed sample disks determined by the Archimedes method with ethanol as the immersion medium are about 97, 96.7, and 96.4% for LATP-30, LATP-50, and LATP-70, respectively. These values are similar to those of the samples prepared by conventional sol-gel or melting-quenching methods [13]. The replacement of Ti^4+^ by Al^3+^ (*x* = 0.4) and the relatively high density of LATP-30 might decrease the *E*a value at the grain boundary, which is beneficial to ion hopping in the electrolyte.

## Conclusions

To summarize, we have developed an evaporation-induced self-assembly (EISA) method for fabrication of nanosized fast lithium-ion conductor LATP (*x* = 0.4) with high ionic conductivity for the first time. This method is simple, applicable for large-scale production, and avoids the difficulty of controlling hydrolysis of titanium salt that conventional sol-gel method has to encounter. The particle size greatly depends on the evaporation rate of the solvent EtOH, which increases gradually with increasing evaporation temperature from 30 to 70 °C. LATP-30 with an average particle size of 70 nm and a relative density of 97% shows a high total lithium-ion conductivity of 3.63 × 10^−4^ S cm^−1^ at room temperature and a low activation energy of 0.286 eV, which will be a promising solid electrolyte for all-solid-state LIBs.

## Abbreviations

EISA: Evaporation-induced self-assembly
LATP: Li_1+*x*_Al_*x*_Ti_2−*x*_(PO_4_)_3_
LIB: Lithium-ion battery
